# Balanced Noise-Evoked Excitation and Inhibition in Awake Mice CA3

**DOI:** 10.3389/fphys.2017.00931

**Published:** 2017-11-21

**Authors:** Ningqian Wang, Xiong Gan, Yun Liu, Zhongju Xiao

**Affiliations:** Department of Physiology, School of Basic Medical Sciences, Southern Medical University, Guangzhou, China

**Keywords:** CA3, acoustic stimulation, balanced inputs, excitation, inhibition

## Abstract

The hippocampus is known as a neuronal structure involved in learning, memory and spatial navigation using multi-sensory cues. However, the basic features of its response to acoustic stimuli without any behavioral tasks (conditioning) remains poorly studied. Here, we investigated the CA3 response to auditory stimuli using *in vivo* loose-patch recordings in awake and anesthetized C57 mice. Different acoustic stimuli in addition to broadband noise such as click, FM sound and pure tone were applied to test the response of CA3 in awake animals. It was found that the wakefulness of the animal is important for the recorded neurons to respond. The CA3 neurons showed a stronger response to broadband noise rather than the other type of stimuli which suggested that auditory information arrived at CA3 via broadband pathways. Finally, we investigated the excitatory and inhibitory inputs to CA3 neurons by using *in vivo* whole-cell voltage-clamp techniques with the membrane potential holding at −70 and 0 mV, respectively. In awake animals, the excitatory and inhibitory inputs CA3 neurons receive induced by noise are balanced by showing stable intervals and proportional changes of their latencies and peak amplitudes as a function of the stimulation intensities.

## Introduction

The hippocampus, participating in learning and memory (Fanselow and Dong, 2010) and spatial navigation (Ito et al., 2015), receives information from multiple sensory modalities (Save et al., 2000; Jeffery, 2007; Ravassard et al., 2013). Auditory information plays a critical role in these hippocampal functions supported by a growing body of evidence from anatomical (Munoz-Lopez et al., 2010), behavioral (Itskov et al., 2012; Miniaci et al., 2016), and electrophysiological (Moxon et al., 1999; Long et al., 2014) studies. The entorhinal cortex (EC) is thought to convey the multi-modal information into the hippocampus (Ahmed and Mehta, 2009; van Strien et al., 2009). Acoustic evoked spikes and c-fos expression were found in the EC (Vinogradov, 1975; Wan et al., 2001). Since the CA3 region of the hippocampus is innervated by the EC (Steward, 1976; Germroth et al., 1989), we have reason to believe that the CA3 should have responses directly induced by acoustic stimulation.

Identifying the basic features of CA3 auditory response will be crucial for understanding the auditory information processing in the hippocampus. Hippocampal acoustic responses have been investigated in anesthetized animals mainly focusing on auditory gating (Miller and Freedman, 1995; Krause et al., 2003; Dissanayake et al., 2008, 2009) and deviations of sound frequency (Ruusuvirta et al., 1995a,b, 2015) or duration (Ruusuvirta et al., 2013). However, the sub-threshold responses induced by sound could be abolished by general anesthesia (Abe et al., 2014b). Therefore, we attempted as a first step of this study to investigate the effects of animal states on the CA3 neuronal responses to broadband noises. Next, we focused on the neuronal responses to different acoustic stimuli, including broadband noise, click, FM sound, and pure tone. We adopted *in vivo* loose-patch recordings to reveal the basic features of the CA3 neurons responding to acoustic stimuli in awake, head-fixed animals. Although, the balance between excitation and inhibition appears to be a common feature of neural circuits in a variety of sensory systems (Okun and Lampl, 2008; Xue et al., 2014; Zhou et al., 2014; Large et al., 2016), Abe et al. (2014a) showed that tone bursts only evoked transient increase of inhibitory post-synaptic conductance in awake CA1 neurons. Since the information flow routes exist mainly from CA3 to CA1 (Deadwyler et al., 1975; Hongo et al., 2015), we attempted to reveal the relationship between the acoustic evoked excitation and inhibition on CA3 neurons by using *in vivo* whole-cell recording techniques and analyzing the changes of their peak amplitude and latency along with stimulation intensity.

## Materials and methods

### General

One hundred and eight C57 mice (female, 4–6 w, 16–18 g, housed with a 12 h light/dark cycle) with normal hearing provided by the Experiment Animal Center of South Medical University were adopted. We handled the animals based on the guidelines provided by the Animal Care and Use Committee of Southern Medical University. Methods for surgery, acoustic stimulation, data acquisition, and processing were similar as previous studies (Tan et al., 2008; Xiong et al., 2013; Zhou et al., 2014). All experimental procedures in this study had been approved by the Animal Care and Use Committee of Southern Medical University.

### Surgical preparation

A week before the recordings, the animals were anesthetized with sodium pentobarbital (60–70 mg/kg, i.p.). The animal's head was fixed to a stereotaxic apparatus by using ear bars and a 1.5 cm-long pole was glued on the dorsal surface of skull with dental cement. The skull over the CA3 (according to the atlas for the mouse brain: −2.30 to −2.90 mm from Bregma, 2.75–3.00 mm lateral to the midline) was opened (1.0 × 1.0 mm) and the dura was removed under a surgical microscope (WPI, Sarasota, FL, USA) and protected from being covered by dental cement. During the following week, the animal was trained to get used to the head fixation using a custom-made holder tightly clamping the pole and running on a rotatable plate.

**Figure 1:**
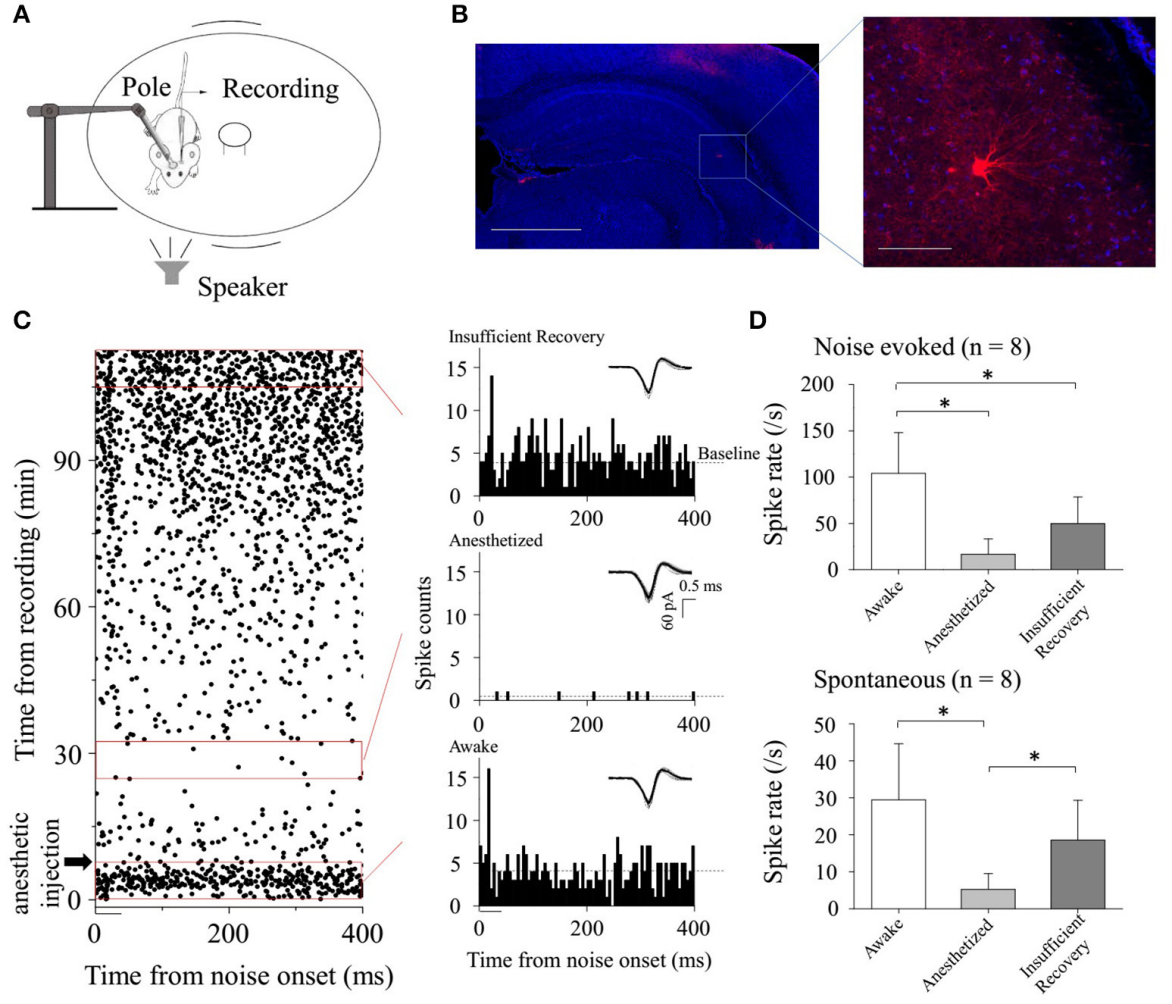
CA3 neuronal responses to broadband noise under different conditions. **(A)** Experimental setup on a rotatable plate for electrophysiological recordings. Pole: used for fixing animal's head. **(B)** Confocal image of hippocampus area (Left, Scale bar: 1,000 μm) and the Biocytin-labeled neuron in CA3 (indicated by the rectangle, Right: Enlarged figure, Scale bar: 100 μm). **(C)** CA3 neuronal spontaneous and noise-evoked responses when the animal was awake, anesthetized and recovery, respectively. Raster plotting (Left) and PSTHs (Right) under different conditions (corresponding to the red rectangles) were shown. The black bar indicated the broadband noise (duration: 50 ms including 5 ms rise/fall time, amplitude: 80 dB SPL). Dashed lines: Baseline of spontaneous discharges. Inserted: imposed waveforms of spikes. **(D)** Neuronal noise-evoked [upper, one-way ANOVA, multiple comparisons: LSD's test, *P* < 0.001 (awake-anesthetized), *P* = 0.003 (awake-recovery), and 0.050 (anesthetized-recovery)] and spontaneous [lower, one-way ANOVA, multiple comparisons: LSD's test, *P* < 0.001 (awake-anesthetized), *P* = 0.061 (awake-recovery), and 0.025 (anesthetized-recovery)] activities changed along with the states of the animals on recorded neurons (*n* = 8. Data obtained from the periods corresponding to the red rectangles in Figure 1C).

For the electrophysiological recordings, the dental cement covering the brain was removed and the awake animal was placed on a rotatable plate while its head was fixed to a stereotaxic apparatus by using the pole glued on the skull (Figure 1A) in a double-walled sound-proof room (temperature maintained at 24–26°C). CA3 neuronal responses to acoustic stimuli in awake animals were recorded. To record the neuronal activity under anesthetized conditions, sodium pentobarbital (60–70 mg/kg, i.p.) was applied to the animal. Vaseline was used to cover the exposed brain during the recordings. The pinnae were maintained as in normal animals. Animal states, awake or anesthesia, were identified on indication by pedal withdrawal reflex as well as the movement of the animal's limbs on the rotatable plate.

### Acoustic stimuli

The acoustic stimuli were generated by a Tucker-Davis Technologies System 3 (TDT3, Tucker-Davis Technologies, Alachua, FL, USA) and delivered via a free-field loudspeaker (ES1, frequency range 2–110 kHz) placed 10 cm away from the front of the animal's head. 1/8 and 1/4 inch microphones (Brüel and Kjaer 4138, 4135, Naerum, Denmark) and an amplifier (Brüel and Kjaer 2610, Naerum, Denmark) were adopted to calibrate the loudspeaker before the experiment.

Broadband noise (0–50 kHz), pure tone (2–64 kHz in 0.1 octave steps), logarithmic frequency modulated (FM) sound (range: 1–32 kHz; speed: 25 octaves/second), and click (0–0.5 kHz) were adopted to test the CA3 neuronal response in this study. The durations of the broadband noise, tone, and FM sound were 50 ms including 5 ms rise/fall time while the click was 0.1 ms long. Different acoustic stimuli with eight sound intensities (20–90 dB SPL in 10 dB steps) were delivered randomly at 1 Hz to the subjects to avoid the adaptation of hippocampal neurons. At least 20 repetitions of each identical acoustic stimulus were given to the subjects.

### Data acquisition

First of all, we tried to determine the range of CA3 for electrophysiological recording according to the atlas for the mouse brain (−2.30 to −2.90 mm from Bregma, 2.75–3.00 mm lateral to the midline, 1.75 to 3.50 mm beneath the brain surface) during the preliminary experiment. The animal's head was fixed as in the atlas for the mouse brain. Then, we placed the electrode into the brain targeting CA3 by using micromanipulator (Siskiyou, OR, USA) following the referenced coordinates and pontamine sky blue was applied iontophoretically. Based on the histological results of the locations of electrode tips, we adjusted the recording location (−2.50 to −2.60 mm from Bregma, 2.90–3.00 mm lateral to the midline, 1.85–2.50 mm beneath the brain surface) to make sure it was in the CA3. All these standards for the CA3 location, including the coordinates and the way of the animal's head fixation, were strictly used in the experiment conducted in this study.

In the present study, loose-patch and whole-cell recordings were performed on the anesthetized and/or awake animals with an Axopatch 700B amplifier (Axon Instruments, Foster City, CA) in the voltage-clamp configuration as previously (Sun et al., 2010; Zhou et al., 2010; Xiong et al., 2013).

A glass pipette filled with artificial cerebrospinal fluid (ACSF in mM: 124 NaCl, 1.2 NaH_2_PO_4_, 2.5 KCl, 25 NaHCO_3_, 20 glucose, 2 CaCl_2_, 1 MgCl_2_, and 0.5% biocytin pH 7.2, Sigma-Aldrich, USA. tip opening: ~1.5 μm, impedance: 5–7 MΩ) was used for loose-patch recordings. The neuronal activities were recorded, amplified (2,000–10,000×), filtered (band-pass: 0.3–3 kHz), and processed by using the TDT3. Single units were isolated based on the spike shape similarity. The shapes and feature spaces (1st to 2nd peak) of spikes (action potentials) during data acquisition were monitored and stored with BrainWare (Version 9.21. Tucker-Davis Technologies, Alachua, FL, USA).

For whole-cell recordings, glass pipettes containing a cesium-based solution (in mM: 125 cesium gluconate, 5 TEA-Cl, 4 MgATP, 0.3 GTP, 10 phosphocreatine, 10 HEPES, 10 EGTA, 2 CsCl, 1.5 QX-314, and 0.5% biocytin, Sigma-Aldrich, USA. pH 7.3, impedance of 5–7 MΩ) were used. The pipette and whole-cell capacitances were completely compensated. The series resistance (20–40 MΩ) was compensated by 50–60% (at 100 ms lag). Signals were low-pass filtered at 2 kHz and sampled at 10 kHz with Clampex (Version 10.2; Molecular Devices, CA, USA). Only neurons with resting membrane potential not higher than −40 mV were investigated. The membrane potentials were held at −70 or 0 mV to study the excitatory or inhibitory inputs, respectively, similar as previous study (Zhou et al., 2010).

The pipettes, controlled by a micromanipulator (Siskiyou, OR, USA), was lowered into the CA3. After the loose-patch recordings, biocytin was applied to the recording site with 20 μA, 15 min by microiontophoresis (Neurophore BH-2, Harvard, USA). An overdose of pentobarbital sodium (100 mg/kg, i.p.) was applied after recording and the animals were perfused transcardially with saline (0.9%) followed by 4% paraformaldehyde in 0.01 M PBS (pH 7.4). Then, we removed the brain and put it in the same fixative overnight at 4°C. For cryoprotection, the brain was immersed in 20% sucrose overnight. Coronal sections for CA3 were cut using freezing microtome (Leica CM 1950, Nussloch, Germany). Sections were washed by 0.01 M PBS (3 × 10 min) and permeabilized with Triton X-100 (0.3%) for 2 h to increase the permeability of antibody. The free-floating sections were incubated with Streptavidin-Cy3 (1:200, Molecular probes, catalog No. 43-4315, USA. at room temperature for 4 h) after PBS rinsing (3 × 10 min). After that, they were washed and transferred to subbed slides and stained with 4′, 6-diamidino-2-phenylindole (DAPI, 0.25 μg/ml) to visualize the cell nucleus. We used confocal microscopes (Nikon, A1R, Japan) to examine the sections to ascertain the location of the recorded neurons. Data obtained from neurons outside the CA3 were discarded. In this study, about 70% of the recorded neurons shown in the results were labeled successfully and all of them were in the CA3. Meanwhile, we think that the remaining neurons (about 30%) should be also in the CA3 because we strictly used the same coordinates as in the preliminary experiment.

### Data analysis and statistics

CA3 neuronal responses to at least 20 presentations of a given sound recorded by using loose-patch recording technique were displayed as peristimulus time histograms (PSTH) and/or raster plotting. A time window (200 ms, starting at the stimulus onset) was selected to calculate the acoustic evoked spike counts (SCs) as well as first spike latencies (FSL, Expressed as Mean ± SD). The mean firing rates of spontaneous activity (baseline) was obtained from a time window (50 ms, starting from 350 ms after the stimulus onset). The FSL was defined as the interval between the acoustic stimulus onset and the time point in the rising phase of the PSTH, where the SCs exceeded the baseline by 2 SDs. The minimal sound intensity eliciting a spike with the probability of 0.1 was defined as minimum threshold (MT) similar as previous work (Keithley and Feldman, 1983).

For the data obtained from the whole-cell recordings, we focused on analyzing the features of excitatory and inhibitory post-synaptic currents (EPSC and IPSC) induced by acoustic stimuli on awake CA3 neurons. The baseline was calculated based on the spontaneous firing within a 50 ms time window ended at the stimulus onset. The onset of acoustic evoked post-synaptic current was defined as the point where the amplitude exceeded the baseline current by 2 SDs in the rising phase (Xiong et al., 2013) while this level (2 SDs exceeding the baseline) was also adopted as zero to calculate the peak amplitude in this study.

For the offline data analysis, we used custom-made MATLAB programs (R2012b; MathWorks, MA, USA). Excel 2007 and OriginPro 8.0 were used to calculate the values of relevant parameters and data fitting and plotting. One-way ANOVA (LSD's Tests for multiple comparisons) was used to compare means and *p* < 0.05 was concerned as significant difference.

## Results

### CA3 neurons only responded to broadband noise in awake state

In the present study, we first attempted to identify the effect of the animal states on the acoustic responses of CA3 neurons (Figure 1B) by using loose-patch recording technique (*n* = 8) in C57 mice. To avoid the adaptation of the CA3 neurons, broadband noises (50 ms, 20–90 dB SPL in 10 dB steps) were applied to the subjects randomly. Neuronal responses to broadband noises (at 80 dB SPL) were extracted. We recorded the neuronal activity before and after the anesthetic injection. Three time windows (0–8, 25–33, and 105–113 min indicated by *red rectangles*; anesthetic injection: 8 min after the onset of recording indicated by *black arrowhead*. Figure 1C) were selected to represent the different animal states on indication by pedal withdrawal reflex and movement on the rotatable plate.

The recorded CA3 neurons responded to the onset of broadband noise when the animals were awake [Figure 1C, Raster plotting (*Left, bottom red rectangle*) and PSTH (*Right, bottom*)]. After the injection of the anesthetic (sodium pentobarbital: 60–70 mg/kg, i.p.), neuronal activities reduced and even had short-term silences [Figure 1C, Raster plotting (*Left, middle red rectangle*) and PSTH (*Right, middle*)]. By comparing the data obtained from corresponding periods (indicated by the *red rectangles*, Figure 1C, *n* = 8), during anesthetized period, both noise-evoked (Figure 1D *upper*, one-way ANOVA, *P* < 0.001) and spontaneous (Figure 1D *lower*, one-way ANOVA, *P* < 0.001) activities were significantly lower than those in awake states. Then, neuronal activities reappeared along with the recovery from anesthesia [Figure 1C, Raster plotting (*Left, top red rectangle*) and PSTH (*Right, top*)]. Insufficient recovery periods (indicated by the *top red rectangles* in Figure 1C) of spontaneous [Figure 1D *lower*, multiple comparisons: LSD's test, *P* = 0.061 (awake-recovery) and 0.025 (anesthetized-recovery)] activities and noise-evoked spikes [Figure 1D *upper*, multiple comparisons: LSD's test, *P* = 0.003 (awake-recovery) and 0.050 (anesthetized-recovery)] were shown. These results suggested that the anesthetic had huge impacts on the responses of the CA3 neurons to acoustic stimulations as well as their spontaneous discharges.

### Neuronal responses to different acoustic stimuli in awake CA3

**Figure 2:**
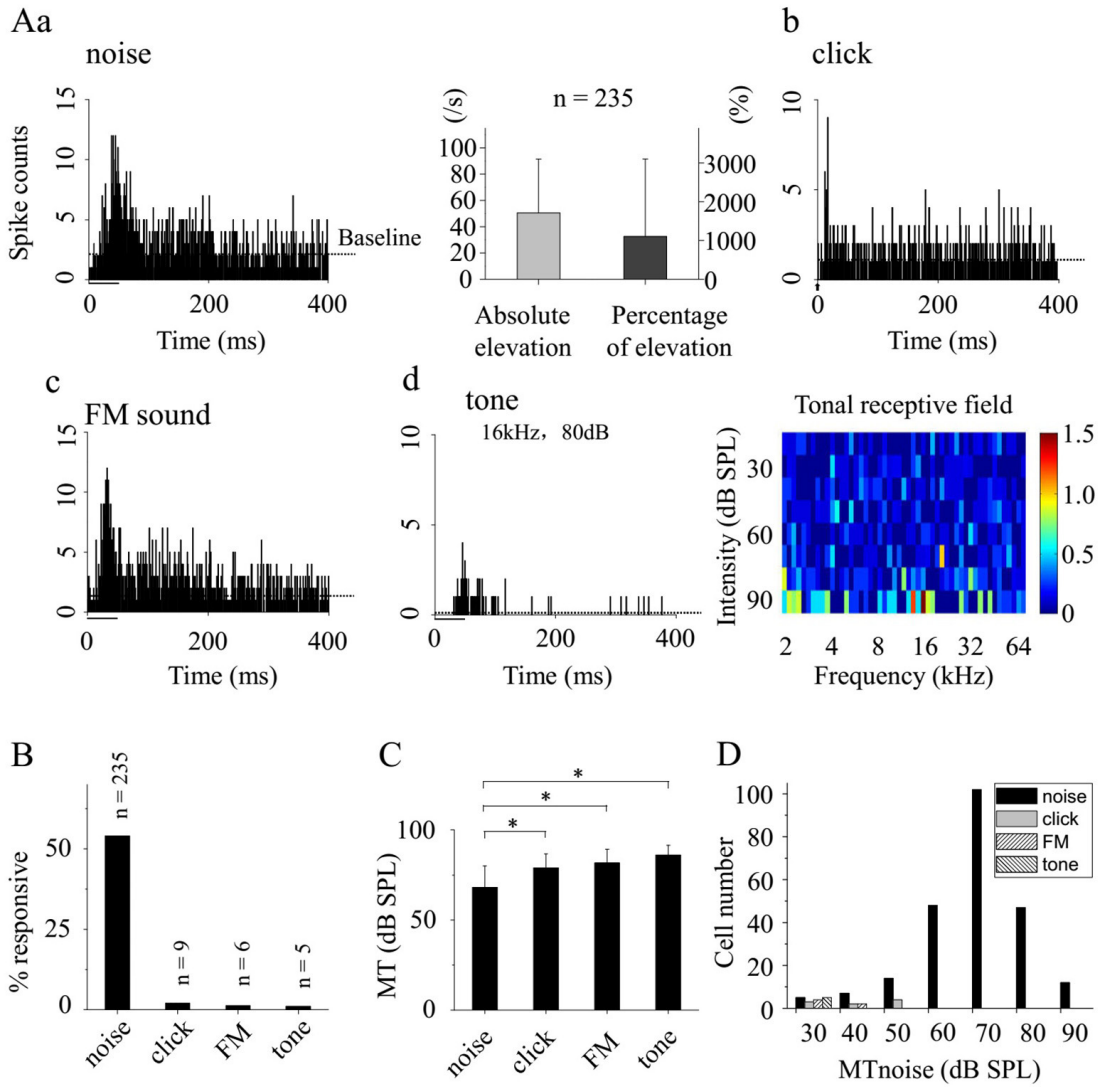
Response features of awake CA3 neurons to different acoustic stimuli. **(A)** PSTHs of the neuronal responses to broadband noise (**a**, left), click **(b)**, FM sound **(c)**, and pure tone (**d**, left). The absolute and percentage elevation over baseline in response to the broadband noise on recorded neurons (*n* = 235) were shown (**a**, right). Meanwhile, the tonal receptive field was shown (**d**, right). The acoustic stimulations were indicated by black bar (noise, click, and tone) or arrowhead (FM sound). **(B)** The probabilities of the recorded CA3 neurons responding to different sounds (noise: *n* = 235, 54.02%, click: *n* = 9, 2.07%; FM sound: *n* = 6, 1.38%; and tone: *n* = 5, 1.15%). **(C)** The MTs of the neurons responding to different acoustic stimuli [noise: 68.04 ± 11.93; click: 78.89 ± 7.82; FM sound: 81.67 ± 7.53; and tone: 86 ± 5.48 dB SPL. One-way ANOVA, LSD's Tests for multiple comparisons. *P* = 0.652 (click-FM), 0.276 (click-tone), 0.540 (FM-tone), 0.007 (noise-click), 0.005 (noise-FM), and 0.001 (noise-tone)]. **(D)** The neuron having click, FM or tone response responded to broadband noise with relative low MTnoise (<60 dB SPL). ^*^P < 0.05.

To determine whether CA3 neurons in the awake animals had preference to different acoustic stimulations, neuronal responses to other acoustic stimuli including click, FM sound and pure tone were also recorded using loose-patch recordings. In awake state, broadband noise (Figures 1C,2Aa, *left*), clicks (Figure 2Ab), FM sound (Figure 2Ac), and pure tone (Figure 2Ad, *left*) could evoke responses (on the same or different neurons). The absolute and percentage elevation over baseline responding to the broadband noise on recorded neurons (*n* = 235) were shown in Figure 2Aa (*right*). Although, neurons had responses to tones of some frequencies at high sound levels (Figure 2Ad: 16 kHz, 80 dB SPL), there were no obvious receptive fields to pure tones on all recorded tone response neurons (*n* = 5. For example: Figure 2Ad, *right*). Neurons responded with much higher probability to broadband noise than to other acoustic stimuli (broadband noise: *n* = 235, 54.02%; click: *n* = 9, 2.07%; FM sound: *n* = 6, 1.38% and tone: *n* = 5, 1.15%, Figure 2B). The recorded neurons responded to click, FM sound and tone with similar MTs [click: 78.89 ± 7.82; FM sound: 81.67 ± 7.53; tone: 86 ± 5.48 dB SPL. One-way ANOVA, LSD's Tests for multiple comparisons. *P* = 0.652 (click-FM), 0.276 (click-tone), 0.540 (FM-tone)] while much lower MTs to broadband noise [68.04 ± 11.93 dB SPL; *P* = 0.007 (noise-click), 0.005 (noise-FM), 0.001 (noise-tone), Figure 2C]. Actually, only the neurons responding to broadband noise with MTs lower than 60 dB SPL might have responses to click, FM sound or tone (Figure 2D).

**Figure 3:**
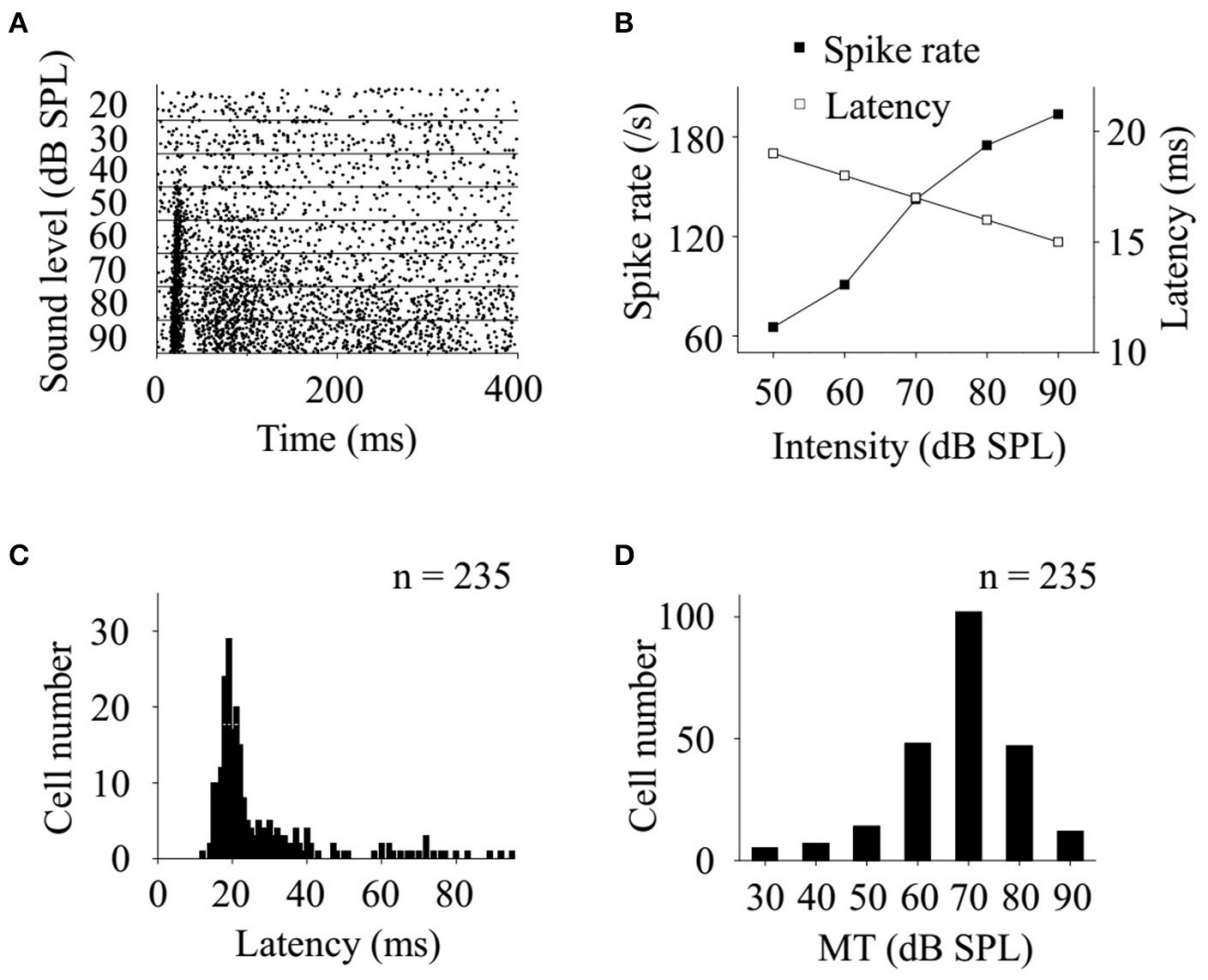
Basic features of CA3 neurons responding to broadband noise in awake animals. **(A)** Raster plotting of neuronal responses to broadband noise at different sound levels. **(B)** When the sound levels increased, the spike rates of the neuronal acoustic responses (filled square) increased while their latencies (open square) decreased. Distributions of response latencies (**C**, at 90 dB SPL) and MTs **(D)** of recorded neurons responding to broadband noise (*n* = 235) were shown.

Since the recorded CA3 neurons showed a preferential response to broadband noise in awake state (Figures 2B–D), we further investigated the basic features of neuronal responses by using broadband noises at different intensities. The SCs of the neuronal responses increased while the FSLs decreased as a function of the sound levels (Figures 3A,B). More than half of the noise response neurons recorded in this study (*n* = 122, 51.91%) had relative short response latencies, 18–25 ms, at a high sound level of 90 dB SPL to the onset of the acoustic stimuli (Figure 3C). The MTs to broadband noise were between 30 and 90 dB SPL while most of them were from 60 to 80 dB SPL (*n* = 197, 83.83%, Figure 3D).

### Excitatory and inhibitory inputs to the recorded awake CA3 neurons

To study the noise-evoked synaptic inputs, *in vivo* whole-cell recordings were adopted and the excitatory and inhibitory inputs were separated by holding the membrane potentials at −70 and 0 mV, respectively (Xiong et al., 2013).

**Figure 4:**
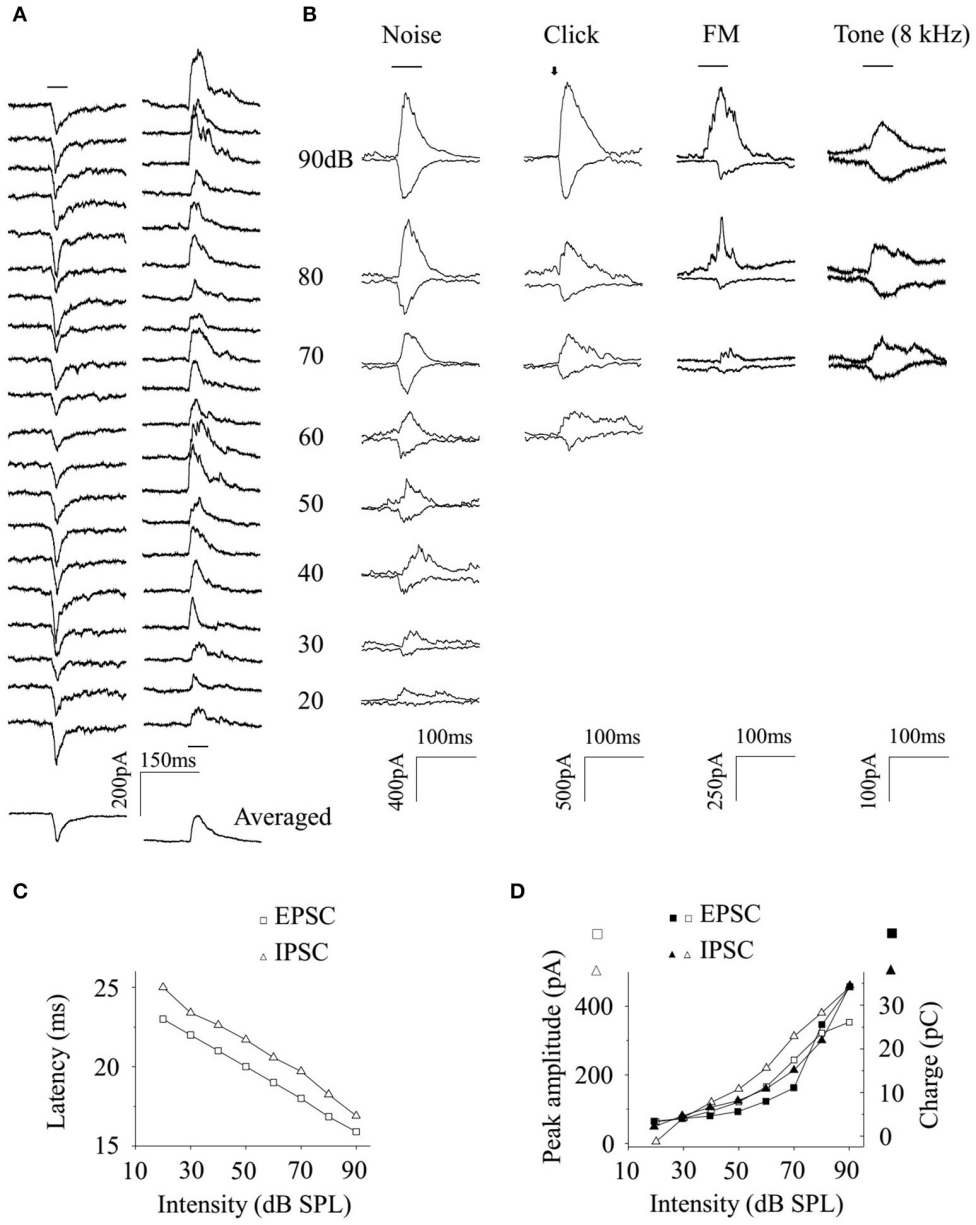
Relationship between the excitatory and inhibitory inputs a CA3 neuron received. **(A)** The EPSCs (Left) and IPSCs (Right) evoked by 20 repetitions of broadband noise given to an awake subject were averaged (Bottom). The averaged EPSC and IPSC evoked by broadband noise, click, FM sound, and pure tone (8 kHz) were plotted together as a function of sound levels **(B)**. The latency **(C)**, peak amplitude (empty, **D**), and charge (filled, **D**) of noise-evoked EPSC (square) and IPSC (triangle) changed in a similar way when the sound intensity changed (Data obtained from Figure 4B. first line). Black bar: the acoustic stimulation.

EPSCs (Figure 4A, *Left*) and IPSCs (Figure 4A, *Right*), induced by 20 repetitions of identical broadband noise, were recorded and averaged (Figure 4A, *Bottom*). The averaged noise-evoked EPSCs and IPSCs were plotted together to different sound levels as in Figure 4B (*first line*). Meanwhile, both excitatory and inhibitory synaptic inputs could also be evoked by click, FM sound or tone (8 kHz) with very low probabilities (only one neuron for each acoustic stimulus. Figure 4B *from second to fourth line*). The basic features of these sub-threshold activities were not compared with those evoked by broadband noise because of the small data size. The latencies (Figure 4C) of noise-evoked EPSCs/IPSCs (Figure 4B, first *line*) decreased and their peak amplitudes and charges (Figure 4D) increased as the sound levels increased, respectively.

**Figure 5:**
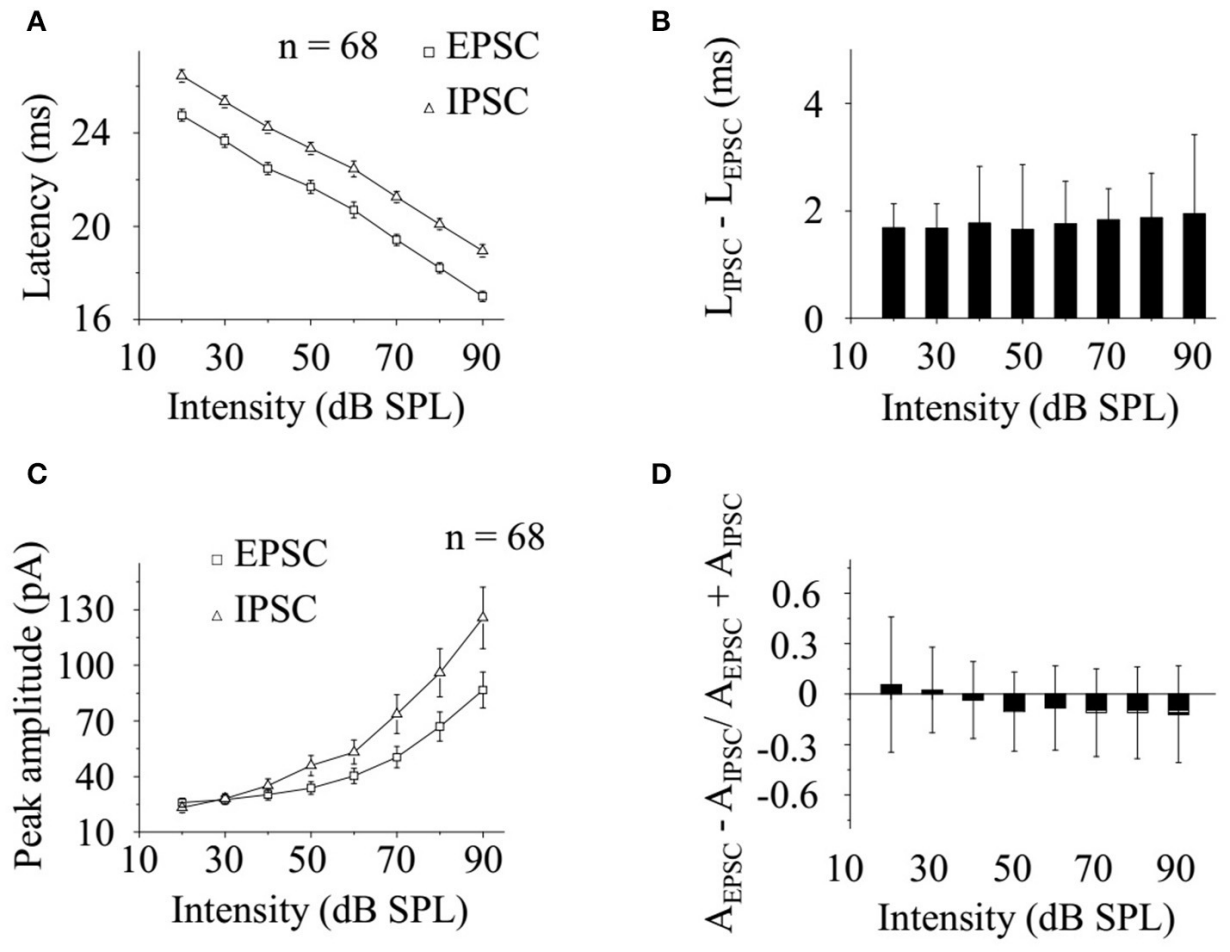
CA3 neurons received balanced excitatory and inhibitory inputs evoked by broadband noise in awake animals. **(A)** The latencies of EPSC (square) and IPSC (triangle) evoked by broadband noise on recorded CA3 neurons (*n* = 68) changed linearly along with the sound levels. **(B)** The difference between the latencies of EPSC and IPSC (L_IPSC_–L_EPSC_) were similar at different sound intensities [One-way ANOVA, between groups (combined): *P* = 0.785; LSD's Tests for multiple comparisons. All *P* > 0.05]. **(C)** The peak amplitudes of noise-evoked EPSC (square) and IPSC (triangle) changed exponentially as a function of sound intensity. **(D)** The normalized peak amplitude difference between EPSC and IPSC at different sound intensities (A_EPSC_ − A_IPSC_/A_EPSC_ + A_IPSC_) had no substantial differences [One-way ANOVA, between groups (combined): *P* = 0.073; LSD's Tests for multiple comparisons. *P* > 0.05 with a few exceptions: *P* = 0.014 (20–80 dB), 0.022 (30–80 dB), 0.023 (40–80 dB), 0.037 (60–80 dB), 0.024 (20–90 dB), 0.032 (30–90 dB), 0.034 (40–90 dB), 0.045 (60–90dB)].

Doing group level analysis on the data obtained from 68 neurons, we found that the latencies of noise-evoked EPSC/IPSC changed linearly as a function of the sound levels (Figure 5A) and the difference between them (L_IPSC_–L_EPSC_) were similar at different broadband noise intensities [One-way ANOVA, between groups (combined): *P* = 0.785; LSD's Tests for multiple comparisons. All *P* > 0.05, Figure 5B]. However, the peak amplitudes of EPSC/IPSC evoked by broadband noise changed exponentially with the stimulation intensities (Figure 5C). The normalized differences between the peak amplitudes of EPSC and IPSC (A_EPSC_ − A_IPSC_/A_EPSC_ + A_IPSC_) at different sound levels had no substantial differences [One-way ANOVA, between groups (combined): *P* = 0.073; LSD's Tests for multiple comparisons. *P* > 0.05 with a few exceptions: *P* = 0.014 (20–80 dB), 0.022 (30–80 dB), 0.023 (40–80 dB), 0.037 (60–80 dB), 0.024 (20–90 dB), 0.032 (30–90 dB), 0.034 (40–90 dB), 0.045 (60–90dB) Figure 5D].

## Discussion

In this study, we tested the hippocampal CA3 neuronal responses to broadband noise in awake or anesthetized animals. The animal wakefulness was important for the recorded neurons responding to broadband noise as well as their spontaneous activities (Figure 1), despite the small sample size (*n* = 8). It is similar as previous results (Abe et al., 2014a,b) that neuronal acoustic responses were largely reduced and even abolished for a short time by general anesthesia. However, anesthetized hippocampal acoustic responses have been reported in many studies (Miller and Freedman, 1995; Ruusuvirta et al., 1995a,b, 2013, 2015; Krause et al., 2003; Dissanayake et al., 2008, 2009). This discrepancy might be mainly due to the different anesthetic, sodium pentobarbital, used in this study. Meanwhile, the difference in experimental animals (rats: Miller and Freedman, 1995; Krause et al., 2003; Dissanayake et al., 2008, 2009; Ruusuvirta et al., 2013, 2015; rabbits: Ruusuvirta et al., 1995a; cats: Ruusuvirta et al., 1995b) might also affect the results. The neuronal sound response in anesthetized animals should be also affected by the acoustic stimulus with (conditioning-testing paradigm: Miller and Freedman, 1995; Krause et al., 2003; Dissanayake et al., 2008, 2009; pitch/duration deviant in tones: Ruusuvirta et al., 1995a,b, 2013, 2015) or without conditioning (our present study) applied to the subjects.

The recorded CA3 neurons had poor responses to acoustic stimuli other than broadband noise by showing low probabilities of supra-threshold (Figure 2B) and sub-threshold events (Figure 4B, only one neuron for each acoustic stimulus). Since both EPSC and IPSC could be evoked by click, FM sound or tone with very low probabilities (only one for each stimulation, Figure 4B), it seems that the poor spiking responses of CA3 neurons to these stimuli might be due to the low percentage of the evoked sub-threshold responses.

The hippocampus is thought to receive auditory information via two major ways. One is concerning the information from the auditory lemniscal pathway. It is processed in the cortical association areas before reaches the hippocampus via the EC (Vinogradov, 1975; Steward, 1976; Steward and Scoville, 1976; Germroth et al., 1989). The other is called non-lemniscal pathway. The auditory information flows to the hippocampus from the brainstem reticular nucleus through the medial septal region (Vinogradov, 1975; Baisden et al., 1984; Moxon et al., 1999). The auditory neurons in non-lemniscal pathway are not tonotopically organized, broadly frequency tuned (Phillips and Irvine, 1979). On all tone response neurons recorded in this study, no clear frequency-intensity tonal receptive field was found (Figure 2Ad, *right*). It is unlikely that there might be an underlying to topographical organization of frequency in the CA3. Our present results that CA3 neurons mainly respond to the broadband noise (Figure 2B) suggest that these auditory information might come to the hippocampus via the non-lemniscal pathway in awake animals. In untrained animals adopted in this study (i.e., acoustic stimuli were applied randomly without any conditioning), it seems likely that the neuronal response is strongest to an unspecific broadband stimulus as the animal does not have developed a specific attention yet.

After the noise-evoked excitatory and inhibitory inputs were separated by using *in vivo* whole-cell voltage-clamp configuration and holding the membrane potentials (Zhou et al., 2010), we found that the excitation and inhibition occurred in a stereotyped temporal sequence by showing stable intervals (Figure 5B). It indicates that excitation and inhibition evoked by broadband noise on CA3 neurons in awake animals are balanced as in previous study (Wehr and Zador, 2003). We also found that both the latency and peak amplitude of the noise-induced excitatory and inhibitory inputs to an awake CA3 neuron changed synchronously along with the sound levels (Figures 4B–D,5). However, the unbalanced, only inhibitory inputs evoked by pure tones via fimbria-fornix pathway were reported on CA1 neurons recently (Abe et al., 2014a) while we found CA3 neurons had poor responses to tones in this study (Figures 2A,B). Since the information flows from CA3 to CA1 directly or via the EC (Deadwyler et al., 1975; Finch et al., 1986; Buzsaki, 1989; Sik et al., 1994; Tamamaki and Nojyo, 1995; Hongo et al., 2015), neural circuits, including hippocampus and EC and even more, might play an important role in the difference of the acoustic evoked inputs in CA3 and CA1.

Both supra- and sub-threshold noise responses on CA3 neurons in awake animals in this study have no intensity selectivity by showing monotonically increases along with the increasing sound levels (Figures 3B, 4D, 5C). The peak amplitudes of the inhibitory inputs were larger than those of the excitatory inputs (Figures 4D, 5C). However, the intervals between excitatory and inhibitory inputs were relative stable in spite of the changes of their latencies as a function of stimulus intensity (Figure 5B). This suggests that the changes of sound levels do not affect the relative timings of excitatory and inhibitory inputs. The monotonic supra-threshold neuronal responses might be explained by the relative unchanged intervals and synchronously changed peak amplitudes of the inhibitory and excitatory inputs (Figures 4B–D,5). The balanced inputs, induced by broadband noise, a CA3 neuron receives in awake mice will help us to understand the hippocampal processing of broadband acoustic information without any conditionings.

## Author contributions

ZX conceived and designed the study. XG and YL performed the experiments. NW analyzed the data and wrote the paper. NW and ZX reviewed and edited the manuscript. All authors read and approved the manuscript.

### Conflict of interest statement

The authors declare that the research was conducted in the absence of any commercial or financial relationships that could be construed as a potential conflict of interest.
